# Editorial: The Tribute of Physiology for the Understanding of COVID-19 Disease

**DOI:** 10.3389/fphys.2021.761644

**Published:** 2021-09-27

**Authors:** Georges Lefthériotis, Susan Wray, Adriana Castello Costa Girardi, Emmanuelle Vidal-Petiot, Matthew A. Bailey, Deborah Schechtman, Nistala Ravi, Denis Noble

**Affiliations:** ^1^Université Côte d'Azur, CNRS, LP2M, Nice, France; ^2^University of Liverpool, Liverpool, United Kingdom; ^3^Laboratory of Genetics and Molecular Cardiology, Heart Institute (InCor), University of São Paulo Medical School, São Paulo, Brazil; ^4^Assistance Publique Hopitaux De Paris, Paris, France; ^5^Centre for Cardiovascular Science, University of Edinburgh, Edinburgh, United Kingdom; ^6^University of São Paulo, São Paulo, Brazil; ^7^University of Missouri, Columbia, KY, United States; ^8^University of Oxford, Oxford, United Kingdom

**Keywords:** editorial, physiology, COVID-19, pandemia, collection

While the specter of the COVID-19 pandemic appears to be gradually receding, the lessons learned from the pandemic are still relevant today. During the year 2021, a collective of physiologists belonging to different national (French, UK, Brazil,...) and European societies (federation of the European Society of Physiology—FEPS), joined forces to make their contribution to the scientific knowledge accumulated during this pandemic.

More than 50 articles have been submitted for publication, and viewed more than 450,000 times, demonstrating the interest of the physiological community in the subject. The articles published in this collection have provided new information or reflections in all areas of physiology, from immunity to respiratory, cardiovascular function, including hemostasis, neurophysiology or even certain related aspects to epidemiology.

The objective of the collection was to provide a better understanding of the interaction between COVID-19 and physiological functions at different stages of organization, from genes to the whole living organism in different disciplinary fields, including cardiovascular, renal, gastrointestinal, endocrine, respiratory and pulmonary, immune and neuronal systems and their physiological functions.

It is important to note that the roles of environmental factors, including age, gender, smoking, metabolic imbalances (e.g., diabetes), as well as immuno-allergic status were taken into account in the selection of articles. The symptoms of the COVID-19 infection have uniquely revealed a richness and diversity focused on a limited time and affecting different populations of cultures and ethnicities. Symptomatology has illustrated interactions and relationships between different physiological functions (e.g., anosmia and neurological symptoms).

The entire invited editorial team is also engaged in the evaluation of interdisciplinary opinions and points of view, as well as data from original work of the different physiological systems targeted by SARS-Cov-2.

The virus is still there with its share of unknowns and uncertainties, and the scientific community too, in an attempt to answer questions from the medical community and human populations in general. The anti-COVID vaccination and its procession of sometimes unusual manifestations is in itself another chapter that opens in the history of this pandemic. There is still significant scientific work to be done in these areas in order to improve our understanding of the mechanisms of this pandemic and to better care for patients and protect us from the next waves.

## Author Contributions

All authors contributed equally to the editorial board of the collection.

## Conflict of Interest

The authors declare that the research was conducted in the absence of any commercial or financial relationships that could be construed as a potential conflict of interest.

## Publisher's Note

All claims expressed in this article are solely those of the authors and do not necessarily represent those of their affiliated organizations, or those of the publisher, the editors and the reviewers. Any product that may be evaluated in this article, or claim that may be made by its manufacturer, is not guaranteed or endorsed by the publisher.

